# Impact of Pre-operative Hemoglobin A1C Level and Microbiological Pattern on Surgical Site Infection After Cardiac Surgery

**DOI:** 10.7759/cureus.11851

**Published:** 2020-12-02

**Authors:** Hani N Mufti, Mayar Jarad, Maryam M Haider, Lein Azzhary, Shahad Namnqani, Imran Husain, Saad Albugami, Wael Elamin

**Affiliations:** 1 Cardiac Surgery, King Faisal Cardiac Center, King Abdullah Medical City, Jeddah, SAU; 2 College of Medicine, King Saud Bin Abdulaziz University for Health Sciences, Jeddah, SAU; 3 Medicine, King Abdullah International Medical Research Center, Jeddah, SAU; 4 Medicine, Ibn Sina National College, Jeddah, SAU; 5 Medicine, Batterjee Medical College, Jeddah, SAU; 6 Medicine, Umm Al-Qura University, Jeddah, SAU; 7 Cardiothoracic Surgery, The Golden Jubilee National Hospital, Glasgow, GBR; 8 Cardiolgy, King Faisal Cardiac Center, King Abdullah Medical City, Jeddah, SAU; 9 Cardiac Surgery, King Faisal Cardiac Center, King Abdulaziz Medical City, Jeddah, SAU

**Keywords:** surgical site infection, cardiac surgery, microbiological pattern, glycated hemoglobin (hba1c)

## Abstract

Background

Surgical site infection (SSI) after cardiac surgery is a major concern. A limited number of studies have addressed the relationship of preoperative glycemic control on the risk of developing SSI after cardiac surgery. We aim to determine the incidence, microbiological pattern, and impact of preoperative hemoglobin A1C (Hgb A1C) on the development of SSI after cardiac surgery.

Methods

This is a single-center retrospective chart review that was performed on adult patients undergoing cardiac surgery from January 2017 to December 2018.

Results

Two hundred and twenty-nine patients underwent 233 procedures. The median age was 60 years; 71% males, 64% were diabetic, and 67% had a Hb A1C above 7% preoperatively. Around 7% of patients developed deep SSI. For patients that developed SSI, 63% had gram-negative bacteria. Hb A1C >7% was not found to be associated with an increased incidence of SSI.

Conclusion

Our results show that there is no apparent relationship between pre-operative Hgb A1C levels and SSI after cardiac surgery. Although we follow a comprehensive SSI perioperative bundle based on international guidelines that advocates using antibiotics to cover gram-positive organisms, it is interesting that the rate of gram-negative organisms in our patients' cohort is unexpectedly high. We believe that adjusting the perioperative antibiotic regimen based on local microbiological patterns seems to be a reasonable and easily achievable target to decrease the incidence of SSI.

## Introduction

Surgical site infection (SSI) after cardiac surgery is a major cause of concern that places a great burden on patients, health care providers, and the health care system, which increases the health care cost by increasing the in-hospital length of stay [1, 2]. SSIs can be categorized into two groups based on the Center of Disease Control (CDC) SSI definition and criteria, which includes at least one of the following criteria within 90 days from the primary surgical procedure: a) organisms cultured from either mediastinal tissue or fluid collected during the operation or by a percutaneous needle aspiration; b) clinical confirmation of mediastinitis during surgery; c) fever, chest pain, wound pain or sternal instability accompanied with either purulent discharge from the mediastinal area; or d) organisms cultured from blood [3]. Unfortunately, this definition is not based on surgical anatomy because it does not take into consideration the depth of infection in relation to the wound edges, so a superficial SSI (defined as wounds that involve the skin and subcutaneous tissue but do not reach the underlying facia of the sternum or the muscle in the harvest site) with positive blood culture is equivalent to a CDC-defined mediastinitis (both are considered CDC-positive SSI), whereas, in fact, these are two totally different entities that carry entirely different outcomes [3, 4]. In this paper, CDC-positive SSI was defined according to CDC SSI criteria as an infection in wounds of any depth (superficial, deep, osteomyelitis or mediastinitis) with at least one positive blood culture containing the same organism as in the surgical site culture or any wound that is beyond the superficial layer (superficial wounds defined as wounds affecting only the skin and subcutaneous tissue). CDC-negative SSI is defined as a superficial SSI without positive blood culture [3].

SSI after cardiac surgery has been associated with major morbidities and up to a 25% risk of mortality [1, 2, 5, 6]. Even though cardiac surgery is classified as a "clean procedure" because there is no involvement of visceral organs, there is still a high incidence of SSI occurring after cardiac surgery. This may be attributed to the patient-related factors and the presence of co-morbidities (diabetes, hypertension, renal diseases, dyslipidemia, obesity) [5, 7-9]. Many studies have found diabetes to be a significant contributor and a risk factor for SSI. Elevated blood glucose levels before, during, or after surgery is a major risk factor for SSI in general [10]. The American Diabetes Association has recommended using Hb A1C level as a method of assessing long-term glycemic control in diabetic patients and the level of Hb A1C to be less than 7% as an indicator of good glycemic control [11]. Also, there is a strong association between elevated Hb A1C and major adverse cardiac events (MACE) after coronary artery bypass grafting. For more precise risk stratification, Hb A1C level before surgery could allow early identification of patients at a higher risk of developing SSI after coronary artery bypass grafting [7]. 

Considering that diabetes has the highest prevalence among all chronic diseases in Saudi Arabia, all efforts should focus on preoperative optimization of glycemic control to assure favorable outcomes of cardiac surgery [12]. A systematic review has found that maintaining blood glucose levels of ≤200 mg/dl throughout all stages of the perioperative period can significantly reduce surgical site infection rates [13]. As the prevalence of diabetes is significantly increasing in Saudi Arabia, not enough studies have addressed the issue of preoperative glycemic control of patients undergoing cardiac surgery [7, 9, 12, 13].

The purpose of this study is to describe the incidence of surgical site infections, the microbiological pattern of causative pathogens and attempt to extract important risk factors with a focus on preoperative glycemic control (assessed by preoperative Hb A1C level) following cardiac surgery in a single center over a two year period from 2017 to 2018.

## Materials and methods

This is a single-center retrospective cohort chart review study that included 229 adult patients, from the beginning of 2017 till the end of 2018, who underwent cardiac surgery at King Faisal Cardiac Center (KFCC), King Abdulaziz Medical City, Ministry of National Guard Health Affairs, Jeddah, Saudi Arabia. Patients were included if they were over 17 years of age and undergone the following procedures: isolated coronary arteries bypass grafts (CABG) surgery, isolated valve surgery, or a combined CABG and valve procedure. Patients were identified using the KFCC cardiac surgery database [14].

Preoperative data included patient demographics (age, gender, weight, height, BMI, body surface area [BSA]), comorbidities such as ischemic heart disease (IHD), diabetes mellitus (DM), DM medications, hypertension (HTN), dyslipidemia (DLP), chronic kidney disease (CKD), stage of renal disease, preoperative dialysis, smoking, chronic obstructive pulmonary disease (COPD), stroke, hospital stay, and invasive procedures before surgery. Preoperative risk stratification was assessed using the European system for cardiac preoperative risk evaluation version II (EURO II SCORE) and Society of Thoracic Surgeons (STS) models, hemoglobin A1c (Hb A1C) when available, preoperative ejection fraction, laboratory workup, and diagnosis. Intraoperative data included operation performed, duration of surgery, use of cardiopulmonary bypass and its duration, and the administration of blood or blood products.

Postoperative data included laboratory workup, need for blood transfusion, SSI based on the CDC categories, wound infection location and type, wound culture, type and number of organisms in the wound culture, blood culture, did the wound require surgery, time from surgery to the diagnosis of SSI, mortality and other complications. SSIs were diagnosed and classified according to the Centers for Disease Control and Prevention criteria [15]. Data was obtained from the hospital's electronic medical record database. This electronic health record is only available for routine patient medical management across the NGHA (National Guard Health Affairs) organization. This means that the data will be available if only done at our health care organization. The study was conducted following the requirements of the local ethics committee with full institutional review board approval from King Abdullah International Medical Research Center (KAIMRC).

Statistical analysis

All statistical analyses were performed using R software, version 3.6.1. (R Foundation for Statistical Computing, Vienna, Austria). We compared the features of patients who developed SSI after surgery to patients who did not. The mean and standard deviation were used for continuous variables that had a normal distribution and were compared using the two-sample t-test or Welch two-sample t-test if the two groups had unequal variance. The continuous variable that was not normally distributed were reported using the median and interquartile range and were compared using the Wilcoxon rank-sum test. Categorical variables were reported as frequencies and percentages and were analyzed by Chi-square or Fisher’s exact test as appropriate. The Kruskal-Wallis test was used for ordinal attributes. The Kaplan-Meier curve was used to compare the effect of different variables on the time of development of SSI up to 90 days, especially preoperative Hb A1C. Univariate and bivariate analysis was done to identify risk factors for the development of SSI after cardiac surgery. All statistical tests were two-tailed, and p-values < 0.05 were considered significant. 

Binary logistic regression was then used to evaluate the influence of several independent risk factors on the development of SSI after cardiac surgery, especially pre-operative Hb A1C. This was reported using the odds ratio (OR) with a 95% confidence interval (CI). The 90 days SSI free probability was calculated from the date of surgery using the Kaplan-Meier method and assessed using the log-rank test. 

## Results

Demographics and clinical characteristics

Two hundred and twenty-nine patients underwent cardiac surgery at KFCC during the study period (January 2017 to December 2018). Out of the 229 patients, 71% were male with a median age of 61 years and body mass index (BMI) of 29 kg/m^2^. Around 66% of patients were diabetic, of which 35% were insulin-dependent. Most patients were hypertensive (~76%) and had a history of IHD (~75%). Only 12.7% of patients had a history of CKD. Around 78% of patients had an ejection fraction (EF) above 40% before the surgery. Almost 50% of the patients had a Hb A1C > 7%. Most patients underwent elective surgery, with 60% of them had an isolated coronary arteries bypass surgery (CABG). The median European system for cardiac preoperative risk evaluation version II (EURO II SCORE) was 1.8, and the median Society of Thoracic Surgeons Adult Cardiac Surgery Risk score was 1 (see Table 1).

**Table 1:** Preoperative characteristics BMI - body mass index; CKD - chronic kidney disease; IHD - ischemic heart disease; IQR - interquartile range; Hgb A1C - hemoglobin A1C; EURO II SCORE - European system for cardiac preoperative risk evaluation version II, STS: Society of Thoracic Surgeons

Patient characteristic	n=229 patients
Age in years, median (IQR)	61 (51-67)
Male gender, n (%)	162 (70.7)
BMI in kg/m^2^, median (IQR)	29 (25.8-32.5)
Hypertension, n (%)	175 (76.4)
Diabetes mellitus, n (%)	150 (65.5)
IHD, n (%)	172 (75.1)
Dyslipidemia, n (%)	185 (80.8)
CKD, n (%)	29 (12.7)
Preoperative dialysis, n (%)	10 (4.4)
Smoking, n (%)	
Active	49 (21.4)
Ex-smoker	437 (16.2)
Non-smoker	143 (62.5)
Chronic obstructive pulmonary disease, n (%)	9 (3.9)
Preoperative stroke, n (%)	17 (7.4)
Preoperative ejection fraction, n (%)	
< 25 %	4 (1.75)
25-40 %	34 (14.85)
41-55 %	108 (47.15)
>55%	71 (31)
Unknown	12 (5.2)
Preoperative Hgb A1C, n (%)	
≤ 7 %	90 (39.3)
> 7 %	108 (47.2)
Unknown	9 (3.9)
Preoperative EURO II Score category, n (%)	
< 5 %	203 (88.7)
≥ 5 %	14 (6.1)
Not Applicable	12 (5.2)
Preoperative STS Mortality Score category, n (%)	
< 5 %	207 (90.4)
≥ 5 %	10 (4.4)
Not Applicable	12 (5.2)

The median length of stay in the intensive care unit was five days, and the median length of in-hospital stay was 14 days. Just over 50% of patients received a postoperative blood transfusion, and only 3.5% of patients developed a postoperative stroke. Overall mortality was around 6% (see Table 2).

**Table 2:** Intra- and postoperative characteristics CABG: coronary artery bypass grafts surgery; AVR - aortic valve replacement; MVR - mitral valve replacement; IQR - interquartile range

Patient characteristic	n=229 patients
Procedure status, n (%)	
Elective	172 (75)
In-house urgent	39 (17)
Emergency	13 (5.6)
Unknown	5 (2.2)
Procedure, n (%)	
CABG	138 (60.3)
AVR	14 (6.1)
MVR	16 (7)
Combined	33 (14.4)
Other	28 (12.2)
Cardiopulmonary bypass time in minutes, median (IQR)	120.5 (95-165.8)
Aortic cross-clamp time in minutes, median (IQR)	79 (59.3-128.3)
Length of stay in the ICU in days, median (IQR)	5 (4-7)
Length of stay in the hospital in days, median (IQR)	14 (10-20)
Postoperative blood transfusion, n (%)	122 (53.3)
Postoperative dialysis, n (%)	13 (5.7)
Postoperative stroke, n (%)	8 (3.5)
Postoperative surgical site infection, n (%)	67 (29.3)
Postoperative mortality, n (%)	15 (6.6)

SSI after cardiac surgery

**Figure 1:**
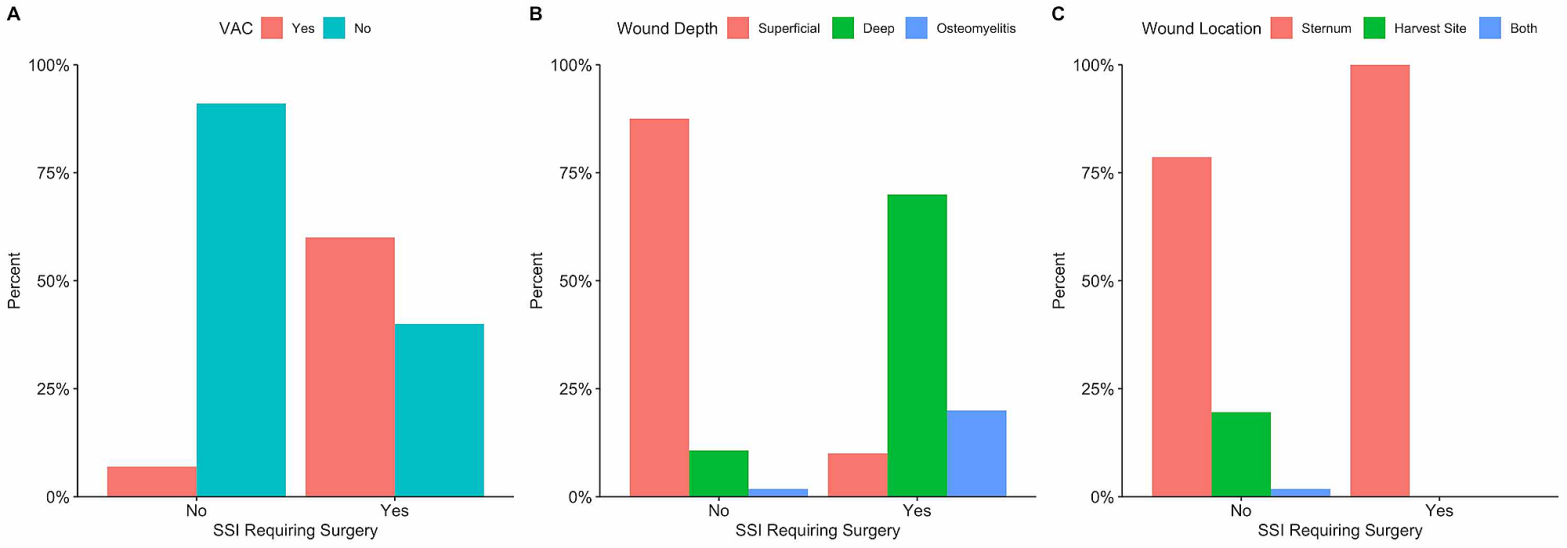
SSI requiring surgery A: Patients categorized based on requiring VAC treatment or not. B: Patients categorized based on the depth of their wounds. C: Patients categorized based on the location of the infection. Superficial: wounds that affect the skin and subcutaneous tissue but do not reach the underlying facia of the sternum or the muscle in the harvest site. Deep: wounds that reach down to the facia of the sternum or the muscle in the harvest site. Osteomyelitis: infection reaching down to or in the boney structure under the wound. SSI - surgical site infection; VAC - vacuum-assisted closure

Out of 229 adult patients, 67 (29.3%) developed a surgical site infection after cardiac surgery. The median time from surgery to the development of SSI was 20 days (IQR: 7 - 32), with just over two-thirds of these patients had a positive wound culture, but only one-third had CDC positive wounds based on the above-mentioned definition [3]. Out of the 67 wound infections, 10 patients (15%) required a vacuum-assisted closure (VAC), with six out of these 10 patients (60%) required a surgical intervention to close the wound. Out of the remaining 57 patients who did not require VAC therapy on their wounds, only four (~7%) required a surgical intervention to close the wound (Figure 1).

Most wound cultures grew gram-negative organisms (63%), and most SSIs were occurring on the midline sternotomy wound (~81%). The most common gram-negative organism isolated from the culture-positive wounds was Escherichia coli (10.5%), followed by Pseudomonas aeruginosa (9%) then Enterobacter (7.5%). Methicillin-sensitive Staphylococcus aureus (MSSA) was isolated from 22.4% of CDC positive wounds, and only 3% had methicillin-resistant Staphylococcus aureus (MRSA) in their wounds. Most patients had only a single organism isolated from their wounds (58.2%). Out of the 67 patients with SSI, 31% had a positive blood culture. Most wound infections were superficial (75%), with only three patients (~5%) having proven osteomyelitis (see Table 3). Patients with culture-positive wounds had similar preoperative characteristics to patients who were culture negative.

**Table 3:** Surgical site infection after cardiac surgery CDC - Center of Disease Control; IQR - interquartile range; SSI - surgical site infection, VAC - vacuum-assisted closure; MRSA - methicillin-resistant Staphylococcus aureus

Patient characteristic	n=67 patients
Time from surgery to infection in days, median (IQR)	20 (7-32)
Surgical site infection CDC category, n (%)	
Positive	21 (31.3)
Negative	46 (68.7)
Surgical site infection location, n (%)	
Leg (saphenous vein site)	11 (16.4)
Sternum	54 (80.6)
Sternum + leg	2 (3)
Surgical site infection depth, n (%)	
Superficial	50 (75)
Deep	13 (19.4)
Osteomyelitis	3 (4.6)
Required VAC, n (%)	10 (14.9)
Wound culture number of organisms, n (%)	
One	39 (58.2)
Two	7 (10.5)
Wound culture organisms type by gram stan, n (%)	
Gram-negative	29 (43.3)
Gram-positive	17 (25.4)
No organism detected	17 (25.4)
Wound culture organism, n (%)	
Staphylococcus aureus	15 (22.4)
Escherichia coli	7 (10.5)
Pseudomonas aeruginosa	6 (9)
Enterobacter	5 (7.5)
Klebsiella pneumoniae	4 (6)
Morganella morganii	2 (3)
MRSA	2 (3)
Serratia marcescens	5 (7.5)
Wound culture second organism, n (%)	
Pseudomonas aeruginosa	4 (6)
Klebsiella pneumoniae	2 (3)
Escherichia coli	1 (1.5)
Candida albicans	1 (1.5)
CDC positive organism in wound, n (%)	
Staphylococcus aureus	8 (38.1)
Escherichia coli	2 (9.5)
Pseudomonas aeruginosa	0 (0)
Enterobacter	3 (14.3)
Klebsiella pneumoniae	3 (14.3)
Morganella morganii	2 (9.5)
MRSA	1 (4.8)
Serratia marcescens	2 (9.5)
CDC negative organism in wound, n (%)	
Staphylococcus aureus	7 (15.2)
Escherichia coli	5 (10.9)
Pseudomonas aeruginosa	6 (13)
Enterobacter	2 (4.4)
Klebsiella pneumoniae	1 (2.2)
Morganella morganii	0 (0)
MRSA	1 (2.2)
Serratia marcescens	3 (6.5)
No organism	21 (45.6)
Postoperative blood culture positive, n (%)	21 (31)
Post-operative SSI requiring surgical intervention, n (%)	10 (14.9)

All CDC positive wounds (n=21) had positive cultures, which grew gram-negative organisms in 57% and 43% gram-positive organisms. Of the CDC negative wounds (n= 46), 37% grew gram-negative organisms in the wounds, 17.4% grew gram-positive organisms, and 46% had no growth. MSSA was present in 38% of CDC positive wounds compared to 27% in CDC negative wounds. The most commonly isolated gram-negative organisms form CDC positive wounds were Enterobacter and Klebsiella pneumoniae (each 14.3%). For CDC negative wounds, the most commonly isolated gram-negative organisms were Pseudomonas aeruginosa (23.1%), followed by Escherichia coli (19.2%) (see Table 3). Patients with CDC negative wound infection were more likely to have preoperative Hb A1C >7% compared to patients with CDC positive wound infection (65% vs. 35%), although it was not statistically significant (p=0.06).

Risk factors for surgical site infection after cardiac surgery

Bivariate analysis was used to assess the relationship among several variables and between patients who developed SSI compared to the ones who did not. We identified two possible predictors of SSI after cardiac surgery in adult patients with a p-value <0.05 (length of stay in the ICU and the length of stay in the hospital; see Table 4).

**Table 4:** Bivariate analysis showing significant predictors of SSI SSI - surgical site infection; ICU - intensive care unit; IQR - interquartile range * Statistically significant with p-value <0.05

Patient characteristic	Total patients (n=229 patients)	SSI: yes (n=67, 29.3%)	SSI: no (n=167, 70.7%)	P-value
Length of stay in the ICU in days, median (IQR)	5 (4-7)	6 (4-7.8)	5 (4-6)	0.038*
Length of stay in the hospital in days, median (IQR)	14 (10-20)	17 (12-24)	13 (10-19)	<0.001*

A univariate logistic regression analysis of these two variables only identified length of stay in the ICU as an independent predictor of SSI after cardiac surgery. Length of stay of more than eight days in the ICU had an odds ratio (OR) 2.2 (95% CI: 1.1 - 4.4, p=0.034), and length of stay of more than 14 days in the hospital had an OR=2.1 (95% CI: 1.2 - 3.9, p=0.013; see Table 5). Hb A1C level of more than 7% before surgery was not associated with increased odds of SSI (OR=1.1, 95% CI: 0.6 - 2.1). Out of the patients who stayed in the ICU for eight days or more, 90% had a prolonged hospital stay > 14 days compared to 47% who stayed for less than eight days in the ICU but had prolonged hospital stay > 14 days (p≤0.001). This might indicate that prolonged ICU stay will lead to a prolonged hospital stay (variables might be correlated), but the opposite is not true. Although prolonged ICU stay seems to be a better predictor, it might be an indirect indicator of the complexity or severity of the primary pathology.

**Table 5:** Univariate logistic regression for significant predictors of SSI SSI - surgical site infection; Hgb A1C - hemoglobin A1C; ICU - intensive care unit; OR - odds ratio; CI - confidence interval * Statistically significant with p-value <0.05

Patient characteristic	OR (95% CI)	P-value
Preoperative Hgb A1C >7%	1.1 (0.6-2.1)	0.83
Length of stay in the ICU >8 days	2.2 (1.1-4.4)	0.034*
Length of stay in the hospital >14 days	2.1 (1.2-3.9)	0.013*

Predictors of SSI within 90 days from surgery

Out of the 67 patients who developed SSI, 66 were diagnosed with an SSI within 90 days from surgery. One patient was diagnosed at 159 days after surgery and presented with chest pain and sternal instability. Osteomyelitis of the sternum was confirmed, which required the removal of two sternal wires and VAC therapy for three weeks to repair the sternum.

**Figure 2:**
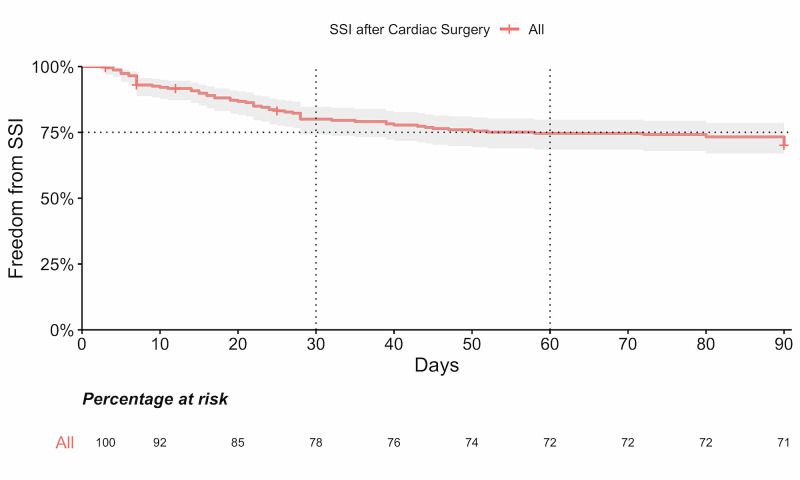
Ninety days freedom from SSI after cardiac surgery in adults SSI - surgical site Infection

Out of the 66 patients who developed SSI within 90 days (28.8% of the total population), 9.2% developed an infection within the first 14 days, 10.8% developed infection between 15-28 days, and 8.8% developed an infection after 28 days (Figure 2).

**Figure 3:**
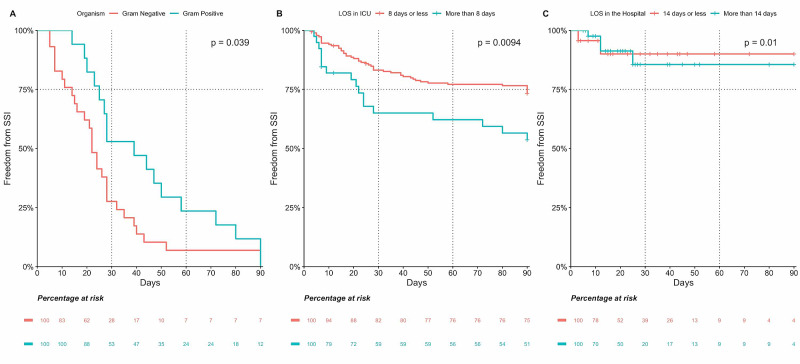
Significant predictors of development of SSI within 90 days from surgery A: Gram stain of the primary organism isolated in the wound culture; B: length of stay in the ICU; C: length of stay in the hospital SSI - surgical site infection; LOS - length of stay; ICU - intensive care unit

Using the log-rank test, three factors were identified to have a significant influence in the development of SSI within 90 days from surgery with a p-value <0.05 (length of stay in the ICU >8 days, length of stay in the hospital >14 days, and having a gram-negative organism identified in the wound culture; see Figure 3). Age >60 years, female gender, BMI>30 kg/m^2^, DM, HTN, DLP, CKD, Hb A1C >7%, procedure status, and type among other variables were not shown to be statistically significant (see Appendix).

## Discussion

In our study, the incidence of all types of SSI after cardiac surgery was quite high (29.3%). Fortunately, deep infection and osteomyelitis were low (5.7% and 1.3%, respectively), which is consistent with the numbers in the literature [3, 5, 16]. Interestingly, in our cohort, there was a higher incidence of gram-negative induced infections compared to gram-positive (43% vs. 25.4%). This is quite different from the published literature where gram-positive induced infections are predominant [2-6, 17]. In a locally conducted retrospective cohort study by Al Majid et al. in Riyadh, with 1241 adult patients who underwent cardiac surgery in a single-center, the incidence of SSI was 3.2% with 45% of infections due to methicillin-susceptible Staphylococcus aureus [5]. They found that neither gender, obesity, diabetes mellitus, non-use of statins, nor CABG surgery were significant predictors of infection. This is consistent with our findings. Table 6 demonstrates the distribution of organisms in patients after cardiac surgery based on gram stain in recent studies published after 2000.

**Table 6:** Cardiac surgery surgical site infection organism distribution of most recent articles published after 2000

Author	Year of publication	% of gram-positive organisms	% of gram-negative organisms
Ridderstolpe et al. [4]	2001	56.5	Unknown
Lepelletier et al. [9]	2013	70	22
Si et al. [2]	2014	53	44
Young et al. [23]	2014	42.1	34.5
Lemaignen et al. [3]	2015	49.7	39
Figuerola-Tejerina et al. [8]	2016	57	43
Majid et al. [5]	2020	57.5	42.5

In our cohort, preoperative glycaemic control of diabetic patients (assessed by Hb A1C levels before surgery) was not found to be a predictor of SSI after cardiac surgery. However, diabetes mellitus is claimed to increase SSI risk, among other complications, especially after valve surgery [7-10, 13, 16, 18]. Several studies have shown conflicting findings [3, 5]. 

Uncontrolled blood glucose levels (BGL) perioperatively were identified in many studies as a major risk factor for surgical complications independent of the presence or absence of DM [19]. A study by Boreland et al. found that sustaining blood sugar levels < 200 mg/dL with a continuous insulin infusion in the perioperative period can help in reducing the incidence of SSI in diabetic patients undergoing cardiac surgery [13]. The Portland Diabetic Project analyzed a large database of cardiac surgery patients (n=5510) who underwent surgery between 1987 and 2005, with hyperglycemia after surgery being a predictor of mortality [19]. Furthermore, Furnary et al. [18] reported that patients with DM whose BGL remained < 150 mg/dL (8.3 mmol/L) had 57% lower risk of mortality and 66% lower risk of deep sternal wound infections compared with diabetic patients who had BGL > 150 mg/dl (8.3 mmol/L). Therefore, several studies aimed to identify the proper target level for controlling BGL for patients with or without diabetes undergoing cardiac surgery. Strict glycaemic control was not recommended by the Normoglycemia in Intensive Care Evaluation-Survival Using Glucose Algorithm Regulation (NICE-SUGAR) trial. The authors reported that patients in the intensive BGL control arm (target 81 to 108 mg/dL) experienced a greater incidence of all-cause mortality at 90 days after surgery versus the conventional BGL target group (target 180 mg/dL or less) [20]. On the other hand, a high BGL > 180 mg/dl was associated with a higher rate of deep sternal wound infections and mortality [18, 21]. According to the 2020 American Diabetic Association recommendations, a target BGL of 140-180 mg/dL was recommended for the critically ill patients and a lower target between 110-140 mg/dL for cardiac surgery patients if the risk of developing hypoglycemia is considered to be low [22].

Interestingly, the most significant SSI predictor in our study was prolonged hospital stay of more than 14 days after surgery. Although this might be misleading, it is most likely a consequence of the SSI rather than its cause [3, 4, 15, 17]. Length of stay in the ICU >8 days was also found to be a significant predictor of developing SSI in our cohort with an OR=2.2 (95% CI: 1.1 - 4.4, p=0.013). This is similar to several other studies showing that the longer the patients stay in the ICU after cardiac surgery, the more likely they develop SSI [1, 3, 5, 8, 15]. However, this might be a consequence of the severity of the patient's primary pathology or complexity of surgery that requires a longer stay in the ICU rather than a direct cause of SSI.

Several external (perioperative) and internal (patient or host-related) factors might affect wound healing [23, 24]. Although cardiac surgical wounds are considered clean wounds [23], the patients undergoing cardiac surgery are at a higher risk of sustaining SSI because of several risk factors (e.g., obesity, DM, peripheral vascular disease, ischemia, smoking, long procedure time, blood transfusion, use of the cardio-pulmonary bypass, etc.) [1-6, 10, 16, 17, 24]. The impact of climate on the rates of SSI is also of importance and can be considered as an external factor [25]. A study by Aghdassi et al. demonstrated a significant increase in the incidence of SSI, particularly with gram-negative organisms, in patients who lived in areas with warmer temperatures (average temperature > 20° C) [25]. The Society of Thoracic Surgeons (STS) did an extensive review of the available literature and came up with two recommendation statements. A class IIA recommendation based on level B evidence, the most commonly prescribed perioperative antibiotics prophylaxis regimen for gram-positive coverage by using first-generation cephalosporins (cefazolin) is reasonable [26]. The addition of an aminoglycoside was a class IIB recommendation and only with vancomycin. When it comes to the duration of antibiotics prophylaxis, the STS recommends antibiotics at least for 24-hours but not to exceed 48-hours [27]. There is some evidence suggesting that widening the antibiotics prophylaxis regimen coverage to include gram-negative organisms with a second or third-generation cephalosporin improves postoperative infections (mainly pneumonia) and all-cause mortality [28]. Some studies even suggest that adding an aminoglycoside to a first-generation cephalosporin provides good coverage for common gram-positive and negative organisms [29]. 

At our institution, we follow a comprehensive pre-, intra-, and postoperative SSI bundle approved by our infection control department, which is based on the recommendations of the Joint Commission International (JCI) “Evidence-Based Principles and Practices for Preventing Surgical Site Infections” [30]. This was applied to the patients included in this cohort. Preoperative preparation includes chlorhexidine gluconate (CHG 4%) soap wash one day before surgery and on the morning of surgery. The hair on the sternotomy and harvesting sites is clipped in the holding bay before entering the operating room. Based on our institutional protocol, patients receive cefazolin in the operating room within 60 minutes of the skin incision and every four hours while on cardiopulmonary bypass, followed by every eight hours for the first 24 hours. Based on the findings of this study and because of the unusually higher incidence of gram-negative organisms isolated from the wounds, we have modified our practice to include an aminoglycoside (gentamycin) to the perioperative antibiotics prophylaxis for adult patients undergoing cardiac surgery in our center (with dose adjustment for the patients with low glomerular filtration rate). Defining microbiological organisms’ local patterns, which colonize cardiac surgical wounds, is of great importance. Obtaining this information can guide local teams to anticipate SSIs, modify infection control policies, or adjust the peri-operative antibiotics prophylaxis accordingly.

Limitations

Some of the limitations include bias attributed to the retrospective and observational nature of the study. The quality of the data may affect the models and their interpretation. The small sample size is another limitation. As for being a single-center cohort, this might limit the generalization of these results. Re-operation of patients with SSI is very subjective, and different surgeons might have different thresholds. Patients who lost follow-up were not included because we don’t know the wound's fate. Finally, under or over-reporting of mild superficial infection might also impact the quality of the data. 

## Conclusions

SSI after cardiac surgery is not an uncommon complication. However, complex wound infections (deep or osteomyelitis) are rare. Preventing SSI after surgery in general and specifically after cardiac surgery is of great importance. Our results show that there is no obvious relationship between preoperative Hgb A1C levels and SSI after cardiac surgery. However, we do have a high incidence of SSI, mainly due to gram-negative organisms. Fortunately, the incidence of deep SSI and osteomyelitis in our cohort is low. Although we follow a comprehensive SSI bundle based on the JCI recommendations that emphasizes antibiotics coverage for gram-positive organisms, we started routinely adding gram-negative coverage based on our population's microbiological pattern. We would recommend adjusting the perioperative antibiotics prophylaxis regimen to include gram-negative coverage, especially in areas where factors favoring the growth of these organisms exist (like high average climate temperature and higher incidence of comorbidities that might affect wound healing).

## Appendices

**Table 7:** Factors influencing 90 days development of SSI after cardiac surgery BMI - body mass index; DM - diabetes mellites; IHD - ischemic heart disease; DLP - dyslipidemia; COPD - chronic obstructive pulmonary disease; Hb A1C - hemoglobin A1C; EF - ejection fraction; CABG - coronary artery bypass grafts surgery; CPB - cardio-pulmonary bypass; ICU - intensive care unit; CDC - Center of Disease Control; SSI - surgical site infection; EURO II SCORE - European system for cardiac preoperative risk evaluation version II; STS - Society of Thoracic Surgeons * Statistically significant with p-value <0.05

Patient characteristic	Number of patients	Observed	Expected	P-value
Age				0.2
≤60 years	105	26	31	
>60 years	124	41	36	
BMI				0.6
≤30 kg/m^2^	133	40	31	
>30 kg/m^2^	96	27	36	
Gender				0.4
Male	162	22	19.2	
Female	67	45	47.8	
DM				0.4
Yes	150	46	43	
No	79	21	24	
IHD				0.4
Yes	172	53	50	
No	57	14	17	
DLP				0.1
Yes	185	58	53	
No	44	9	14	
CKD				0.3
Yes	29	6	8.9	
No	200	61	58.1	
Smoking, active				0.2
Yes	49	11	15.2	
No	180	56	51.8	
COPD				0.7
Yes	9	2	2.7	
No	220	65	64.3	
Hb A1C				0.3
≤7%	89	23	26.9	
>7%	109	37	33.1	
EF				0.1
≤40%	38	15	10.4	
>40%	191	52	56.6	
Procedure status				0.1
Elective	172	55	49.7	
Others	57	12	17.3	
Procedure type				0.8
CABG	138	41	40	
Others	91	26	27	
CPB time				0.3
≤120 minute	99	33	28.7	
>120 minute	107	30	34.3	
Cross clamp time				0.1
≤80 minute	104	37	30.5	
>80 minute	102	26	32.5	
Length of stay in the ICU				0.009*
≤8 days	186	49	56.4	
>8 days	39	17	9.61	
Length of stay in the hospital				0.01*
≤14 days	104	22	32.5	
>14 days	39	17	9.61	
EURO II Score				0.8
≤ 5%	203	59	59.3	
> 5%	14	5	4.37	
STS mortality score				0.5
≤ 5%	207	62	60.7	
> 5%	10	2	3.3	
CDC SSI Category				0.9
Negative	46	46	46.4	
Positive	21	21	20.6	
Wound culture				0.2
Negative	21	21	17	
Positive	46	46	50	
Organism type				0.04*
Gram-negative	29	29	22.6	
Gram-positive	17	17	23.4	
Mortality				0.2
Yes	15	2	4.9	
